# Mechanism of a novel *Bacillus subtilis* JNF2 in suppressing *Fusarium oxysporum* f. sp. *cucumerium* and enhancing cucumber growth

**DOI:** 10.3389/fmicb.2024.1459906

**Published:** 2024-11-13

**Authors:** Fan Yang, Xin Wang, Huayan Jiang, Qiuju Yao, Shen Liang, Weiwei Chen, Gongyao Shi, Baoming Tian, Abeer Hegazy, Shengli Ding

**Affiliations:** ^1^Institute of Vegetable, Henan Academy of Agricultural Sciences, Graduate T&R Base of Zhengzhou University, Zhengzhou, Henan, China; ^2^School of Agricultural Sciences, Zhengzhou University, Zhengzhou, Henan, China; ^3^Institute of Horticulture, Henan Academy of Agricultural Sciences, Zhengzhou, Henan, China; ^4^National Water Research Center, Shubra El Kheima, Egypt; ^5^Henan Agricultural University, Zhengzhou, Henan, China

**Keywords:** *Fusarium oxysporum* f. sp. *cucumerinum*, *Bacillus subtilis*, biocontrol bacteria, growth promotion, whole genome sequence

## Abstract

Cucumber *Fusarium* wilt caused by *Fusarium oxysporum* f. sp. *cucumerium* (*FOC*), is a prevalent soil-borne disease. In this study, *Bacillus subtilis* JNF2, isolated from the high incidence area of cucumber *Fusarium* wilt in Luoyang, demonstrated significant inhibitory effects on *FOC* and promoted cucumber seedling growth. The biocontrol mechanism of strain JNF2 were elucidated through morphological observation, physiological and biochemical experiments, and whole genome sequence analysis. Pot experiments revealed an 81.33 ± 0.21% control efficacy against *Fusarium* wilt, surpassing the 64.10 ± 0.06% efficacy of hymexazol. Seedlings inoculated with JNF2 exhibited enhanced stem thickness and leaf area compared to control and hymexazol-treated plants. Physiological tests confirmed JNF2’s production of indole-3-acetic acid (IAA), siderophores, and hydrolytic enzymes, such as β-1,3-glucanase, amylase, and protease, which inhibited *FOC* growth and promoted plant development. Genome analysis identified genes encoding antimicrobial peptides and hydrolases, as well as a novel glycocin synthetic gene cluster. These findings underscore *B. subtilis* JNF2’s potential as a biocontrol agent for sustainable cucumber cultivation.

## 1 Introduction

Cucumber is a globally cultivated vegetable crop with significant economic importance (Che and Zhang, 2019). *Fusarium* wilt, caused by *Fusarium oxysporum* f. sp. *cucumerium* (*FOC*) is a common soil-borne disease affecting cucumber plants. The symptoms include neck constriction, wilting, lodging, and curling of leaves in the later stage, and the near-ground side begins to gradually wither until the top (Huang et al., 2019). Therefore, this disease will lead to a certain degree of reduction in cucumber yield (Sun et al., 2017). The pathogen infects the roots of host plants, colonizing the vascular tissue (Di Pietro et al., 2003). It can invade flowers and young melons, causing necrosis and damage, yellowing and wilting of leaves, and finally leading to plant death (Zhou et al., 2017). The traditional methods of controlling cucumber *Fusarium* wilt include biological, chemical, and agricultural approaches (Zhao et al., 2014). While chemical control can temporarily suppress pathogens in the soil in a short period of time, long-term use may harm soil and lead to pathogen resistance (Guo et al., 2023). Agricultural control, though effective, is labor-intensive and not always comprehensive (Rahman et al., 2021). Therefore, new changes have taken place in human attitudes toward these methods of prevention and treatment (Shafi et al., 2017), and concurrently, there is a shift toward exploring more sustainable and environmentally friendly biological control methods. Existing studies have shown that microbial agents can be directly applied to the soil to inhibit the growth and reproduction of pathogenic microorganisms, thereby reducing the incidence of diseases (Ghosh et al., 2017).

Biological control involves using one organism to manage another organism, offering benefits such as environmental friendliness, sustainable development, and safety compared to non-biological control methods. Various microbial species, including *Bacillus* (Dan et al., 2010), *Pseudomonas* (Tziros et al., 2007), and *Trichoderma* (Mo et al., 2012), had proven effective in combating *Fusarium* wilt (Wang et al., 2021). Chen et al. (2017) identified *Bacillus cereus* Y3F in seaweed samples, demonstrating a 50.46% control rate against cucumber *Fusarium* wilt. Research indicates that many biological controls not only inhibit the pathogen growth but also enhance plant growth through mechanisms like nutritional competition, antibacterial compound synthesis, growth-promoting compound release, and plant defense response induction (Du et al., 2017). Chu et al. (2023) observed that the use of *Bacillus* as a biocontrol agent can boost cucumber growth and reduce cucumber *Fusarium* wilt incidence. Similarly, Liang et al. (2013) found that actinomycete CT205 promotes cucumber growth, and achieves a 51.85% control rate against cucumber *Fusarium* wilt. *Bacillus* is a kind of widely existing in nature, which has the characteristics of fast reproduction, simple nutritional requirements and a broad antibacterial spectrum, making it effective against plant diseases, including soil-borne on Lan et al. (2023). *Bacillus subtilis*, a thermophilic, aerobic, spore-forming Gram-positive bacterium with rod shape, is heat, drought, ultraviolet light resistant, produces endospores (Obagwu and Korsten, 2003). It has a wide range of inhibition across plant parts, making it an ideal biocontrol agent. Previous studies have demonstrated that *Bacillus subtilis* can colonize plant roots and rhizosphere soil, and by competing with pathogenic bacteria for nutrients, secreting of substances that hinder the growth and development of these pathogens, and induce plant defense enzyme activity. *Bacillus subtilis* helps plant resist diseases like *Fusarium* wilt (Huang et al., 2010). However, different strains of *Bacillus subtilis* may have varying mechanisms for inhibiting *Fusarium* wilt pathogens, necessitating further research.

Many recent studies have examined the mechanism of action of *Bacillus subtilis* against plant pathogens. However, the current research on *Bacillus subtilis* is still mainly focused on the aspects of screening, identification and biocontrol ability, while there are fewer reports on the biocontrol mechanism of *Bacillus subtilis* against cucumber *Fusarium* wilt and its colonization ability. *Bacillus subtilis* JNF2 is a new strain with efficient biocontrol ability against the pathogen *FOC*-FJH36 obtained by screening in this study, which not only inhibits the growth of the pathogenic fungus but also promotes the development of cucumber seedlings. In order to investigate how strain JNF2 in cucumber inter-root soil can control cucumber *Fusarium* wilt, this study focused on determining the content of relevant metabolites and enzyme activities of strain JNF2 to investigate its mechanism of fungal inhibition and growth promotion; whole genome resequencing and prediction of gene function of strain JNF2 were performed, to discuss whether there are any differences in disease resistance mechanism between strain JNF2 and other strains, and to preliminarily explore the mechanism of biocontrol against the disease. These results will provide a reference for the future validation of functional genes related to the resistance of strain JNF2 to cucumber *Fusarium* wilt, and will provide a theoretical basis and guidance for the application in the field of biocontrol.

## 2 Materials and methods

### 2.1 Isolation of soil bacteria

*Fusarium oxysporum* f. sp. *cucumerinum* (*FOC*-FJH36) was isolated and preserved from the Institute of Vegetable, Henan Academy of Agricultural Sciences. The bacterial strain JNF2 was isolated and screened from an area with a high incidence of cucumber *Fusarium* wilt in Beiwang Village, Luolong District, Luoyang City, Henan Province, China, and cultured on Luria Bertani plate.

### 2.2 Screening of antagonistic bacteria

The plate confrontation method was used for screening (Li et al., 2018). The *FOC*-FJH36 fungus was served as an indicator strain, and a 5 mm diameter puncher was used to obtain a cucumber *Fusarium* wilt fungus cake from the edge of the pathogen colony edge. The fungus cake was then inoculated in the center of the potato dextrose agar (PDA) plate, with the bacteria inoculated on both sides of the fungus cake 25 mm away from the pathogen. The control group consisted of a PDA plate inoculated only with *FOC*-FJH36. After 7 days of incubation at 30°C, the growth of the pathogenic bacteria was observed. Each treatment was replicated three times to assess the inhibitory effect of JNF2 on fungi growth by comparison with the control group.

### 2.3 Observation on the inhibitory effect of JNF2 on the mycelial growth of *FOC*-FJH36

Mycelia measuring no more than 3 mm^3^ were excised from the antagonistic PDA plate. The samples underwent a gentle rinse with phosphate buffered Saline (PBS) (2.7 mM KCl, 2.0 mM KH_2_PO_4_, 137 mM NaCl, 10 mM Na_2_HPO_4_, pH 7.4), followed by immersion in an electron microscope fixative at room temperature for 2 h, before storage at 4°C. Subsequently, the fixed samples were subjected to three washes with 0.1 M phosphate buffer (PB) (2.7 mM KCl, 2.0 mM KH_2_PO_4_, 10 mM Na_2_HPO_4_, pH 7.4) for 15 min each time. A 1% osmic acid solution was prepared using 0.1 M PB (pH 7.4) and applied to the samples for fixation in the darkness at room temperature for 2 h. Post fixation, the samples underwent three 15-min rinses with 0.1 M PB. Dehydration was carried out using ethanol with sequential concentration of 30, 50, 70, 80, 90, 95, and 100% for 15-min each time, followed by a 15-min soak in isoamyl alcohol. The samples were then dried in a Quorum K850 critical point dryer, affixed tightly to conductive carbon film with double-sided adhesive and subjected to placed on the sample stage of 30 s of gold spraying using the Hitachi MC1000 ion sputtering instrument. Observation and imaging were performed with the Hitachi SU8100 scanning electron microscope (Hitachi Advanced Technology Co., Tokyo, Japan).

### 2.4 Determination of antibacterial and growth-promoting substances of strain JNF2

The study evaluated the growth-promoting and biocontrol effects of strain JNF2 were evaluated through measurement of the β-1,3-glucanase, protease, and amylase activity, as well as IAA and siderophore production ability of JNF2 (Yang et al., 2024).

For β-1,3-glucanase activity, JNF2 was inoculated in the middle of β-1,3-glucanase agar plate (peptone 1.0 g, glucose 0.05 g, sodium chloride 0.5 g, yeast extract 0.5 g, Congo red 0.01 g, agar 18.0 g, 1,000 mL sterile distilled water), and cultured at pH 7 and 30°C for 48 h. The white and clear area around the strain JNF2 indicated that the strain had β-1,3-glucanase activity.

For the amylase activity, JNF2 was inoculated in the middle of the starch agar plate (10.0 g soluble starch, 10.0 g tryptophan, 5.0 g NaCl, 5.0 g beef extract, 18.0 g agar, 1,000 mL sterile distilled water) under the same conditions of IAA production. Lugol iodine solution (1% iodine solution, 2% potassium iodide w/v) was added to the starch agar medium to make it evenly spread on the plate. After standing for one minute, a colorless area around the colony was observed, indicating that the strain had amylase activity.

The strain JNF2 was inoculated in the center of skim milk agar medium containing 0.1223 g CaCl_2_, 5.0 g NaCl, 10.0 g peptone and 18.0 g agar per 1,000 mL of sterile distilled water. The culture was maintained at pH 7 and 30°C for 48 h, resulting in the development of a transparent area around the colony, indicating the strain’s protease activity.

Similarly, a single strain JNF2 was inoculated in the center of L-tryptophan liquid medium composed of 3.0 g beef extract, 5.0 g NaCl, 10.0 g peptone, 0.5 g·L^−1^ L-tryptophan and 18.0 g agar per 1,000 mL of sterile distilled water. The culture was maintained at pH 7.2 and 30°C for 48 h. After centrifugation at 14,000 × g for 10 min, 1 mL of the supernatant was admixed with 2 mL Salkowski dye and left at room temperature for 30 min. The color change from yellow to orange indicated the production of IAA.

JNF2 was inoculated in the center of Chrome Azurol S (CAS) blue agar (10 mL 20% sucrose solution, 30 mL 10% acid-hydrolyzed casein, 1 mL 1 mmol·L^−1^ CaCl2, 5 mL 0.1 mol·L^−1^ phosphate buffered saline (pH = 6.8), 50 mL CAS staining solution, 18 g agar powder, 1,000 mL double distilled water, pH 7.0), and cultured at 30°C for 48 h. If an orange halo is generated, it indicates that JNF2 can produce siderophores.

### 2.5 Potted disease prevention test of strain JNF2

Pot experiments were conducted to evaluate the biocontrol effect of JNF2 bacteria on cucumber *Fusarium* wilt. The suspension of *FOC*-FJH36 was prepared by inoculating *Foc*-FJH36 on a PDA plate and culturing at 30°C for 5 days. Fungal cakes were taken from the colony edge of the pathogen with a 5 mm puncher and cultured in 100 to 140 mL of potato dextrose broth (PDB). After incubation with shaking at 30°C and 180 rpm for 3 days, the cultures were filtrated and diluted to a pathogen spore suspension at 1 × 10^6^ spores·mL^−1^. The bacterial strain JNF2 was also prepared by culturing in LB medium at 37°C, 180 rpm for 24 h, and finally followed 4,000 × g of centrifugation for 5 min to make a suspension with LB medium to 1 × 10^8^ cfu·mL^−1^.

In this study, the cucumber seeds with strong, full and uniform size in Bojie 616 were selected and germinated. Cucumber seeds were immersed in 75% ethanol (v/v) for 30 s, followed 5 times of rinse with sterile water, and then immersed in warm water at 55°C for 10 min. Subsequently, the seeds were immersed in sterile water for 4 h. After thorough washing, the seeds were neatly arranged on the gauze and cultured in a constant temperature incubator at 28°C. When the seeds were exposed to white, the germinated cucumber seeds were sown in a nursery pot (40 holes) equipped with a sterile substrate. After 10 days of sowing, the cucumber seedlings reaching to the second true leaf fully expanded, the cucumber seedlings of the same size were screened and transplanted to a flowerpot with a diameter and height of 10 cm and 400 g sterile matrix.

Four experimental groups were designed in this study. Experimental group 1 involved irrigation with 20 mL of sterile distilled water three times, followed by irrigation with a 20 mL *FOC* pathogen spore suspension 24 h later. Experimental group 2 consisted of irrigating the roots three times with 20 mL 0.1% Hymexazol, followed by irrigation with 20 mL *FOC* spore suspension after 24 h. In experiment group 3, the roots were irrigated three times with 20 mL JNF2 suspension, followed by irrigation with 20 mL *FOC* spore suspension after 24 h. Experimental group 4 involved irrigating cucumber seedlings with 20 mL of JNF2 suspension three times, once every 2 days. Each treatment was replicated five times, with each replicate containing 5 pots of plants. The treated pots were placed under greenhouse conditions with a temperature range of 14°Cto 25°C and a light–dark cycle of 16 h light and 8 h dark.

Various parameters such as plant height, stem diameter, leaf area, chlorophyll content, fresh weight and dry weight of aboveground and underground parts were measured on the 20 th and 40 th day after inoculation, and the strong seedling index was calculated (Yang et al., 2023). *Fusarium* wilt grading standards were used to assess the health of the plants, with grades ranging from that grade 0 is normal growth, asymptomatic; grade 1 is cotyledon etiolation, but not wilting; level 2 is cotyledon wilting; level 3 is cotyledon and true leaf wilting or plant dwarfing; level 4 is complete death of the plant (Chen et al., 2022).

The seedling index, disease index and control effect formula are shown below for data analysis.

Seedling index = (stem diameter/plant height + root dry weight/shoot dry weight) × whole plant dry weight.

Disease index = *Σ* (disease grade × total plant number of disease grade) / (maximum disease grade × total plant number) × 100 (Chen et al., 2023).

Control effect (%) = (control group disease index-treatment group disease index) /control disease index × 100% (Zheng et al., 2011).

### 2.6 Determination of defense enzyme activity in cucumber leaves

On the 20th day of treatment, plant leaves of were collected to determine enzyme activity. In this study, polyphenol oxidase (PPO) test box, superoxide dismutase (SOD) test box, phenylalanine ammonia lyase (PAL) test box, catalase (CAT) and lipoxygenase (LOX) test box were used to detect the activities of polyphenol oxidase, superoxide dismutase, phenylalanine ammonia lyase, catalase and lipoxygenase (He et al., 2021). The procedures and methods followed the instruction provided with the kit.

### 2.7 DNA extraction, genome sequencing, and assembly of strain JNF2

The strain JNF2 was inoculated into LB liquid medium and cultured in a shaker at 37°C, 150 rpm for 18 h. Subsequently, the DNA of strain JNF2 was extracted using the Mini BEST bacterial genomic DNA extraction kit Ver3.0. A DNA insertion fragment of approximately 10 kb was then prepared and sequenced utilizing the PacBio Sequel II system and Frasergen in Wuhan, Hubei, China. The reads obtained were *de novo* assembled and sequenced using HGAP4 (Chin et al., 2013) and Canu (Koren et al., 2017) software, following the three generations of HiFi reads were aligned to the assembled genome using the minimap2 (V2.15-r905) software to analyze the coverage depth of the genome. The assembled whole genome sequence of JNF2 was deposited in NCBI GenBank under accession number CP065789.1. Finally, the software Circos was employed to generate the genome circular map of strain JNF2 (Krzywinski et al., 2009).

### 2.8 Genome annotation of strain JNF2

Initially, the genetic elements were predicted, and the coding gene of strain JNF2 was identified by Glimmer (v3.02) (Delcher et al., 2007), tRNAscan-SE (v2.0.9) was used to identify tRNA (Lowe and Eddy, 1997), the rRNA gene was identified by RNAmmer (v1.2) (Lagesen et al., 2007), and the remaining RNA was identified by Infernal’s (v1.1.4) (Nawrocki and Eddy, 2013) cmscan program to align the genome of JNF2 strain to the Rfam database for identification. Secondly, the blastp command of the software diamond (v2.0.9.147) (Buchfink et al., 2015) was used to compare the protein sequences of the predicted genes of the strain JNF2 to the NCBI Non-Redundant Protein Database (NR), Swiss-Prot database, Clusters of Orthologous Groups database (COG), and Kyoto Encyclopedia of Genes and Genomes database (KEGG). The alignment parameters were set to E value 1e^−5^, and the hit with the highest score was selected as the final annotation result. Gene Ontology database (GO) annotation was performed using ID Mapping (20210616) and go-basic. Obo (v2021-09-01).

Furthermore, the blastp parameters of diamond (v2.0.9.147) software were used to align the protein sequence of the predicted gene of strain JNF2 to the CARD-v3.1.4 database, PHI database, VFDB SetA database, TCDB database, E value 1e^−5^, and the hit with the highest score was selected as the final annotation result.

### 2.9 Analysis and identification of strain JNF2

The whole genome sequences of the following strains were extracted from the NCBI database: *Bacillus subtilis* 73, *Bacillus subtilis* CV16, *Bacillus subtilis* JCL16, *Bacillus cereus* J1, *Bacillus cereus* E33L, *Bacillus velezensis* VJH504, *Bacillus velezensis* H208, *Bacillus pumilus* ZB201701, *Bacillus pumilus* EB138, *Bacillus licheniformis* T5, *Bacillus licheniformis* BL1202, *Bacillus mycoides* Gnyt1, *Bacillus mycoides* PAMC 29434, *Bacillus amyloliquefaciens* X030 and *Bacillus amyloliquefaciens* 35 M. Among them, the corresponding GenBank IDs of these strains were: CP045826.1, CP062497.1, CP054177.1, NZ_AP022921.1, NC_006274.1, CP131928, NZ_CP097359.1, NZ_CP029464.1, NZ_CP081199.1, CP124852.1, CP017247.1, NZ_CP020743.1, NZ_CP136372.1, NZ_CP040672.1 and NZ_CP082278.1. Their average nucleotide identity with JNF2 was calculated using the ANI calculator (Yoon et al., 2017).

### 2.10 Analysis of CAZymes enzyme and secondary metabolic genes of strain JNF2

CAZy is a professional database dedicated to carbohydrate enzymes, which includes related enzyme families involved in carbohydrate degradation, modification, and biosynthesis. In this study, dbCAN2 (Zheng et al., 2023) and HMMER (v3.1b2) (Finn et al., 2011) were used to align the predicted protein sequences in the strain JNF2 genome with the carbohydrate-active enzyme (CAZy) database, and the E value threshold set to 1e^−15^. Gene clusters associated with secondary metabolite synthesis were identified by comparing protein-coding genes in the strain JNF2 with the CAZy database through antiSMASH (v7.0.0) (Blin et al., 2019).

### 2.11 Collinearity analysis of strain JNF2

The chromosomes of *Bacillus subtilis* JNF2, *Bacillus subtilis* 73, *Bacillus subtilis* JCL16 and *Bacillus subtilis* CV16 were globally aligned using the software Mauve (v2.3.1) (Darling et al., 2004). The collinearity diagram of JNF2 and three *Bacillus subtilis* strains was created using TBtools (v1.0697) (Chen et al., 2020) to visually represent the comparison results.

### 2.12 Statistical analysis

One-way analysis of variance (ANOVA) was conducted in SPSS (v21.0) to analyze the experimental data. The comparisons of mean value were performed using the Duncan multi-range test with statistical significance set at *p* ≤ 0.05.

## 3 Results

### 3.1 Biocontrol and growth-promoting characteristics of JNF2 strain

**Figure 1:**
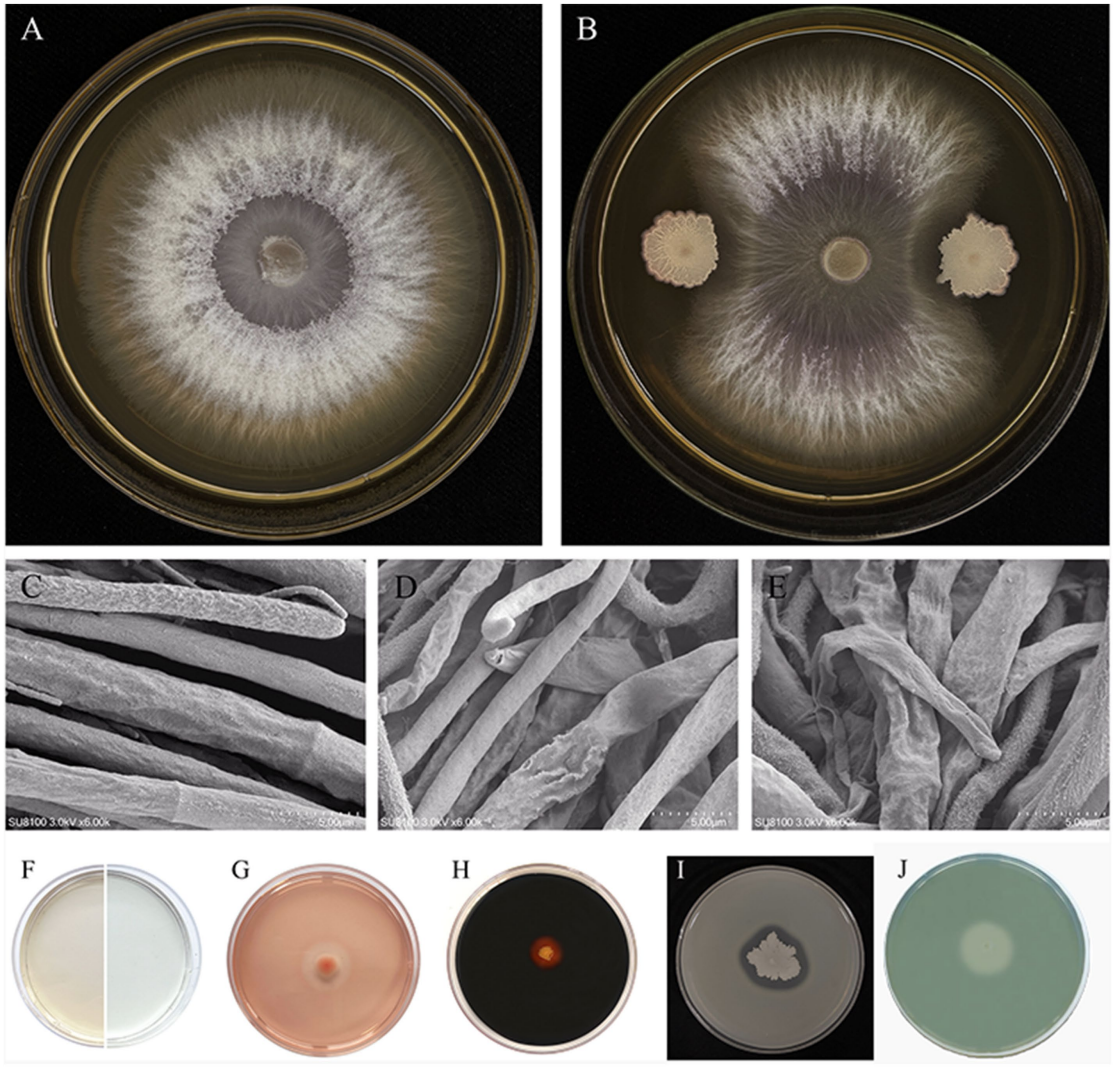
Biocontrol effect of strain JNF2. (A) *Foc*-FJH36 colony morphology without JNF2 strain on PDA plate; (B) Colony morphology of *Foc*-FJH36 containing JNF2 strain on PDA plate; (C) The morphological development of *Foc*-FJH36 was normal; (D) JNF2 strain caused the contraction of *Foc*-FJH36 mycelium; (E) JNF2 strain caused *Foc*-FJH36 to shrink and wilt; (F–J) From left to right, the results of indole-3-acetic acid (IAA), β-1,3-glucanase activity, amylase activity, protease activity and siderophore of JNF2 strain were detected.

The antagonistic activity of the JNF2 strain against the cucumber *Fusarium* wilt pathogen *FOC*-FJH36 was examined through a plate confrontation test. After 5 days of confrontation, the result demonstrated that JNF2 exhibited a significant inhibitory effect on *FOC*-FJH36, with an inhibition rate of 86.52% and an antagonistic zone of 8 mm (Figures 1A,B). Scanning electron microscopy revealed that the pathogenic mycelium in the control group displayed normal characteristics such as smooth and straight (Figure 1C). In contrast, the pathogenic mycelium in the confrontation plant showed abnormal features like shrinkage, wilting and shriveling (Figures 1D,E).

The study also showed that the strain JNF2 had the ability to secrete β-1, 3-glucanase (Figure 1G), amylase (Figure 1H) and protease (Figure 1I), which act on the fungal cell wall to antagonize pathogens. Moreover, through the discoloration of L-tryptophan and CAS medium, it indicates that strain JNF2 can produce indole-3-acetic acid (Figure 1F) and siderophore (Figure 1J), that is, it has a positive effect on plant growth and development.

### 3.2 The activity of JNF2 strain against *Fusarium* wilt in cucumber plants

**Figure 2:**
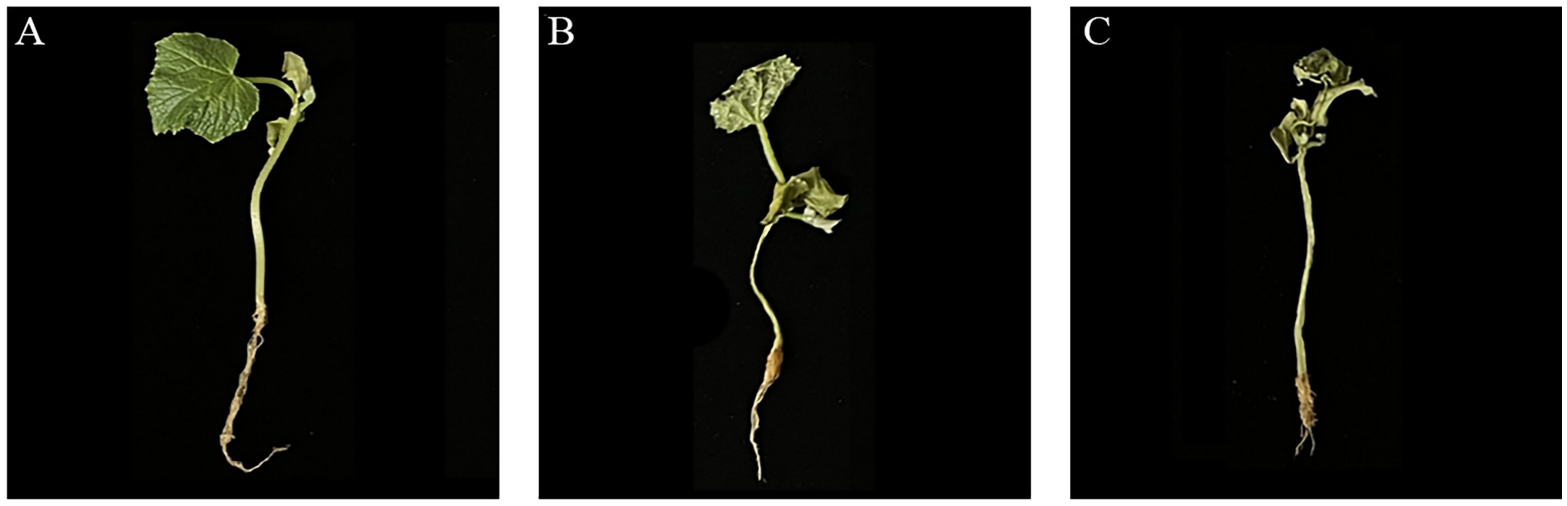
The control effect of strain JNF2. (A) The control effect of *B. subtilis* JNF2; (B) The control effect of Hymexazol; (C) Cucumber seedlings inoculated with *FOC*.

The results showed that the incidence of *Fusarium* wilt in cucumber seedlings treated with JNF2 strain was significantly reduced at 20 days after inoculation with *FOC*. On the 20 th day after inoculation, there were significant differences in the incidence, disease index and biocontrol effect of different treatments. Among them, JNF2 strain could significantly reduce the incidence of *Fusarium* wilt caused by *FOC* (Figures 2A,C). The cucumber seedlings inoculated with JNF2 strain had thicker stems and larger leaf area than those of the control and Hymexazol treated cucumber plants and the root system. Compared with the control (Figures 2A,B), the morbidity and disease index under this treatment were reduced by 77.59 and 83.05%, respectively (Table 1). The data showed that the relative control effect of JNF2 strain was higher than that of chemical fungicide Hymexazol.

**Table 1:** The pot control effects of strain JNF2.

Treatment	Morbidity (%)	Disease index	Control effect (%)
*FOC*	96.21 ± 0.36^a^	72.26 ± 0.31^a^	
*FOC* + 0.1% Hymexazol	27.68 ± 0.11^b^	21.13 ± 0.21^b^	64.10 ± 0.06
*FOC* + JNF2	21.56 ± 0.71^c^	12.25 ± 0.04^c^	81.33 ± 0.21

The numerical value is mean ± standard error (SE), *n* = 3. There was a significant difference between the mean values of different letters in the column (*p* < 0.05). Within the same column, different letters (a-c) indicate significant differences at *p* < 0.05 level.

### 3.3 Effect of JNF2 strain on defense enzyme activity of cucumber seedlings

After 20 days of inoculation with strain in cucumber seedlings, the activities of LOX, PAL, CAT, PPO and SOD were significantly increased compared to the control group. Among them, the activities of LOX, PAL and CAT in cucumber seedlings inoculated alone with *FOC* were higher than those inoculated with JNF2. Except for SOD, the activities of other four defense enzymes in cucumber seedlings treated with *FOC* + JNF2 were significantly higher than those in other treatment groups (Table 2).

**Table 2:** Effect of JNF2 strain on defense enzyme activity of cucumber.

	LOX (U/g)	PAL (U/g)	CAT (U/g)	PPO (U/g)	SOD (U/g)
CK	459.85 ± 33.31^d^	11.71 ± 1.13^e^	106.08 ± 3.78^e^	22.85 ± 1.61^d^	105.21 ± 4.06^c^
*FOC*	1,545.24 ± 42.43^b^	21.27 ± 2.52^c^	152.59 ± 2.75^c^	74.03 ± 4.5^b^	159.57 ± 1.46^b^
*FOC* + 0.1% Hymexazol	636.59 ± 18.05^c^	28.18 ± 0.88^b^	184.74 ± 7.32^b^	43.76 ± 2.62^c^	195.91 ± 11.01^a^
*FOC* + JNF2	3,867.2 ± 41.36^a^	38.08 ± 4.18^a^	233.91 ± 6.20^a^	197.23 ± 8.03^a^	125.62 ± 2.77^c^
JNF2	646.42 ± 9.55^c^	20.08 ± 2.47^d^	127.23 ± 4.18^d^	69.54 ± 3.99^b^	162.78 ± 1.08^b^

The numerical value is mean ± standard error (SE), *n* = 3. There was a significant difference between the mean values of different letters in the column (*p* < 0.05). Within the same column, different letters (a-e) indicate significant differences at *p* < 0.05 level.

### 3.4 Growth-promoting effect of JNF2 strain on cucumber seedlings

**Figure 3:**
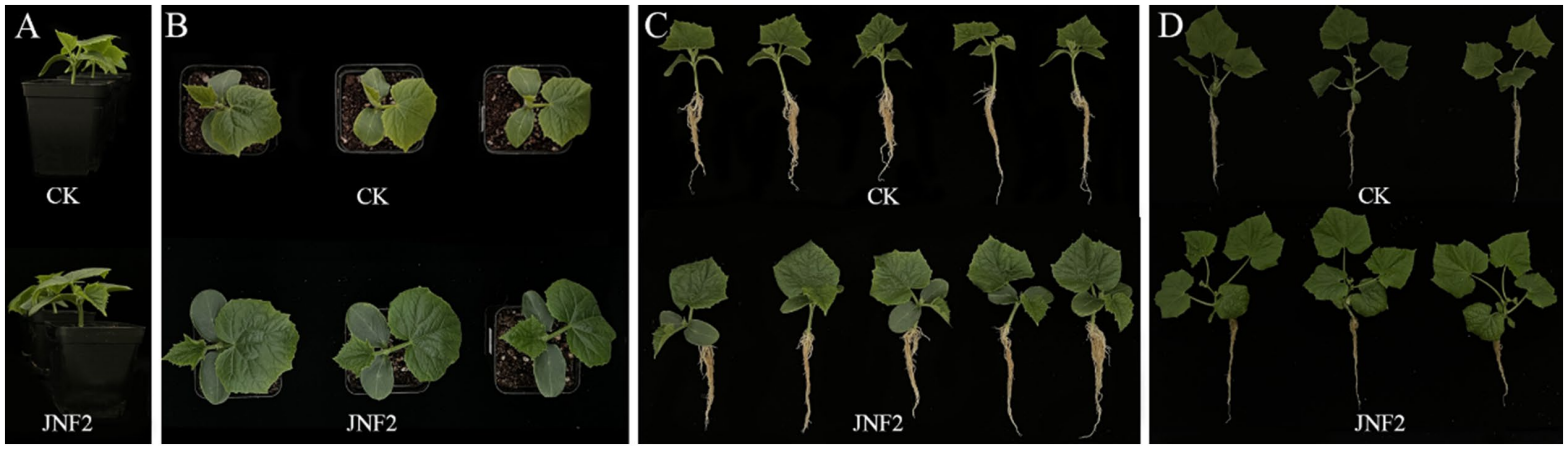
Growth-promoting effect of strain JNF2. (A–C) Cucumber seedlings were cultured for 20 days; (D) Cucumber seedlings were cultured for 40 days.

**Figure 4:**
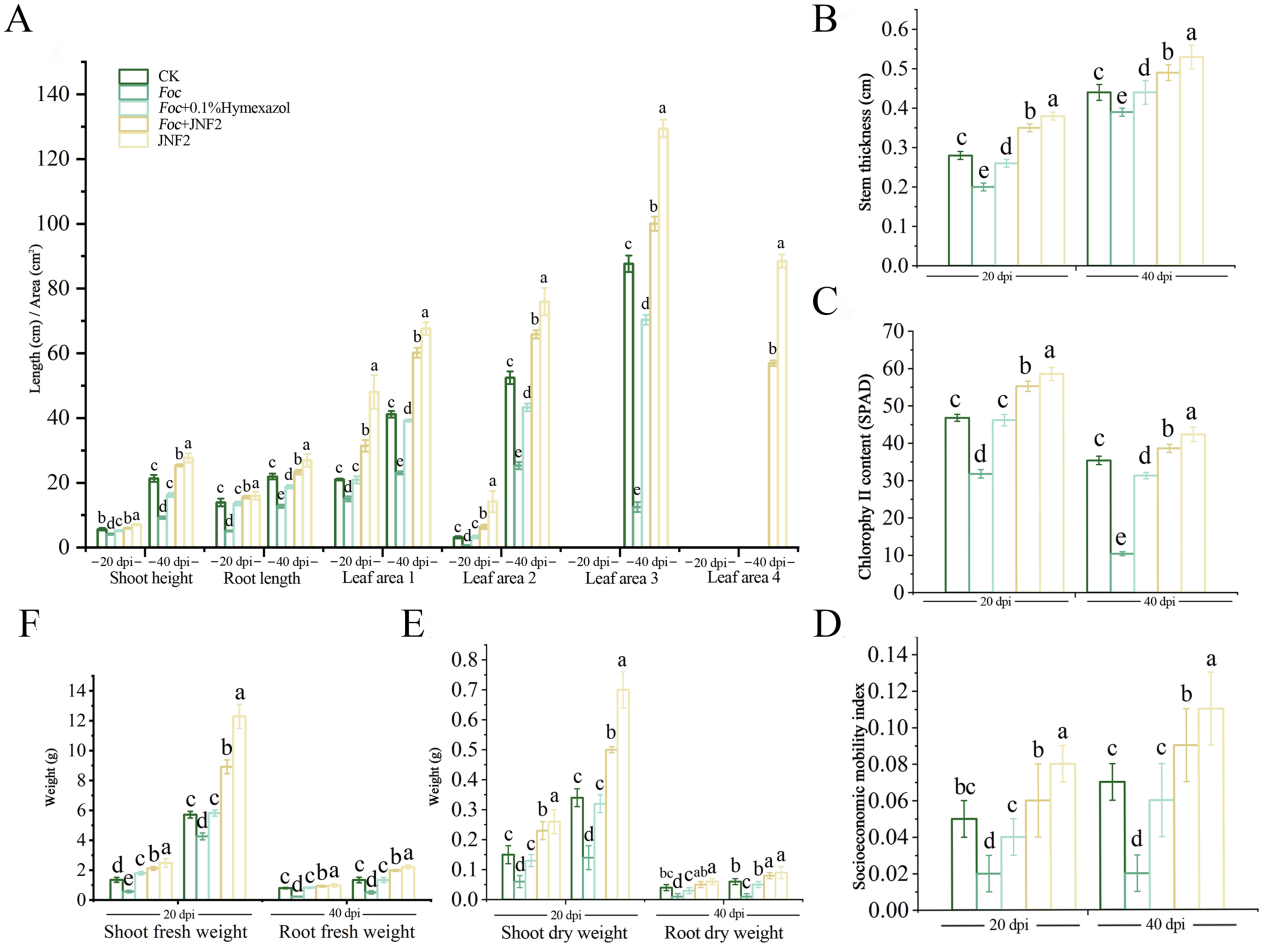
The growth-promoting effect of strain JNF2 on cucumber plants at 20 and 40 days. (A) Plant height, root length and leaf area of cucumber seedlings at 20 and 40 days post-inoculation (dpi); (B) The stem diameter of cucumber seedlings at 20 and 40 dpi; (C) The chlorophyll content of cucumber seedlings at 20 and 40 dpi; (D) The strong seedling index of cucumber at 20 and 40 dpi; (E) The root and shoot dry weight of cucumber seedlings at 20 and 40 dpi; (F) Root and shoot fresh weight of cucumber seedlings at 20 and 40 dpi after inoculation.

On the 20 th and 40 th day after inoculation, the JNF2 strain had a significant positive effect on the growth of cucumber compared with the control group (Figures 3A–D). The results show that the cucumber seedlings treated with the strain were significantly higher than other treatment groups in plant height, stem diameter, root length, leaf area, chlorophyll content, aboveground fresh weight, aboveground dry weight and underground fresh weight (Figures 4A–F). At 20 dpi, the increase of the second true leaf area reached the maximum, with an increase of 345.45% (Figure 4A; Supplementary Table S1). At 40 dpi, the increase of the fourth true leaf area with JNF2 bacterial solution was the largest with the average area of 88.5 mm^2^, while less than 5 mm^2^ in the control group (Figure 4A; Supplementary Table S2).

### 3.5 Whole genome sequencing and assembly of JNF2 strain

**Figure 5:**
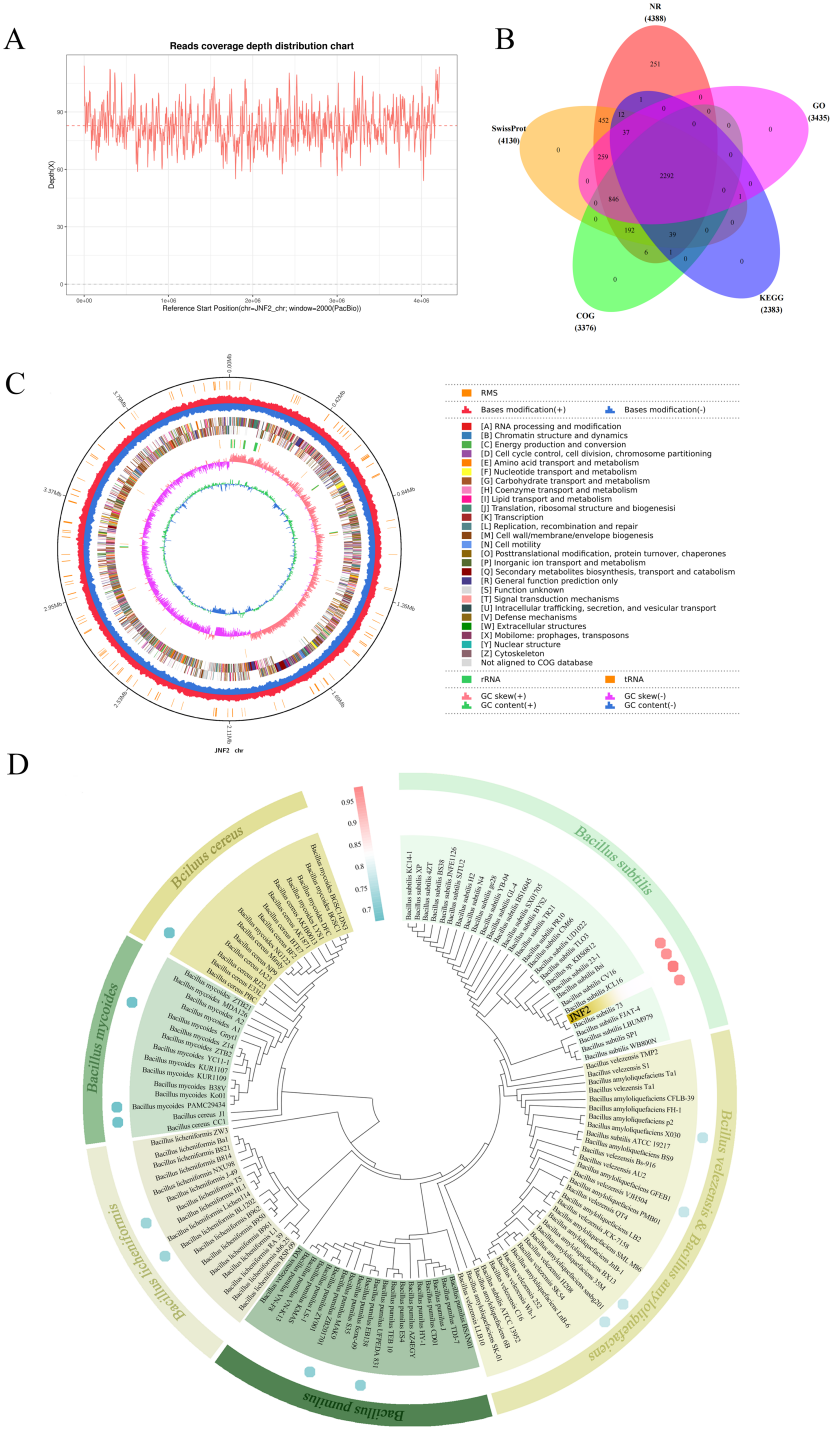
Basic information of whole genome sequencing, assembly, functional annotation and identification of strain JNF2. (A) Read the coverage depth distribution map; (B) Statistical analysis of common and unique annotations of strain JNF2 basic database; (C) Genome circle map of strain JNF2; (D) Species identification of strain JNF2.

This study utilized PacBio RSII sequencing technology in conjunction with NGS sequencing technology to construct two sequencing libraries of the second and third generations, and the whole genome sequencing of JNF2 strain was completed efficiently and accurately to explore its disease resistance mechanism. A total of 604,648 subreads were produced by DNA sequencing of the strain. The average length of the subreads was 11,106.61 bp, and the N50 was 11,969 bp. The average coverage depth of the genome was 82.77 × (Figure 5A), and the assembled genome sequence was deposited in GenBank (accession number CP065789.1).

The circular genome of *B. subtilis* JNF2 is shown in Figure 5C. The genome of *B. subtilis* JNF2 is composed of 4,215,642 bp circular chromosomes, with the GC content at 43.51%. A total of 4,424 protein-coding genes were predicted, covering 88.39% of the genome, and the remaining 218 genes were tRNA (86 genes), rRNA (30 genes) and other non-coding RNAs (102 genes). The protein sequences of the predicted genes were annotated to the corresponding database (E value = 1e^−5^), and 4,388 (99.19%), 4,130 (93.35%), 3,376 (76.31%), 2,383 (53.87%), and 3,435 (77.64%) protein-coding genes showed matching in NR, SwissProt, COG, KEGG and GO databases, respectively (Figure 5B). Among them, the whole genome of strain JNF2 was annotated in the Swissprot database, including a large number of genes associated with siderophore biosynthesis, such as *feuA, feuB*, and *feuC* (Supplementary Table S3).

The phylogenetic tree was constructed by the 16S rDNA sequence alignment of strain JNF2 and the comparison of the whole genome of the strain with other strains. As shown in Figure 5D, JNF2 strain is clustered with *Bacillus subtilis*, indicating that it has an intimate relationship with *Bacillus subtilis*. ANI analysis compared the genetic correlation between JNF2 and 4 strains of *B. subtilis*, 2 strains of *B. amyloliquefaciens*, 2 strains of *B. velezensis*, 2 strains of *B. pumilus*, 2 strains of *B. licheniformis*, 2 strains of *B. mycoides* and 2 strains of *B. cereus*. Among them, the ANI value between JNF2 and *B. subtilis* 73, *B. subtilis* JCL16 and *B. subtilis* CV16 was more than 98%, exceeding the 96% correlation threshold (Supplementary Table S4).

**Figure 6:**
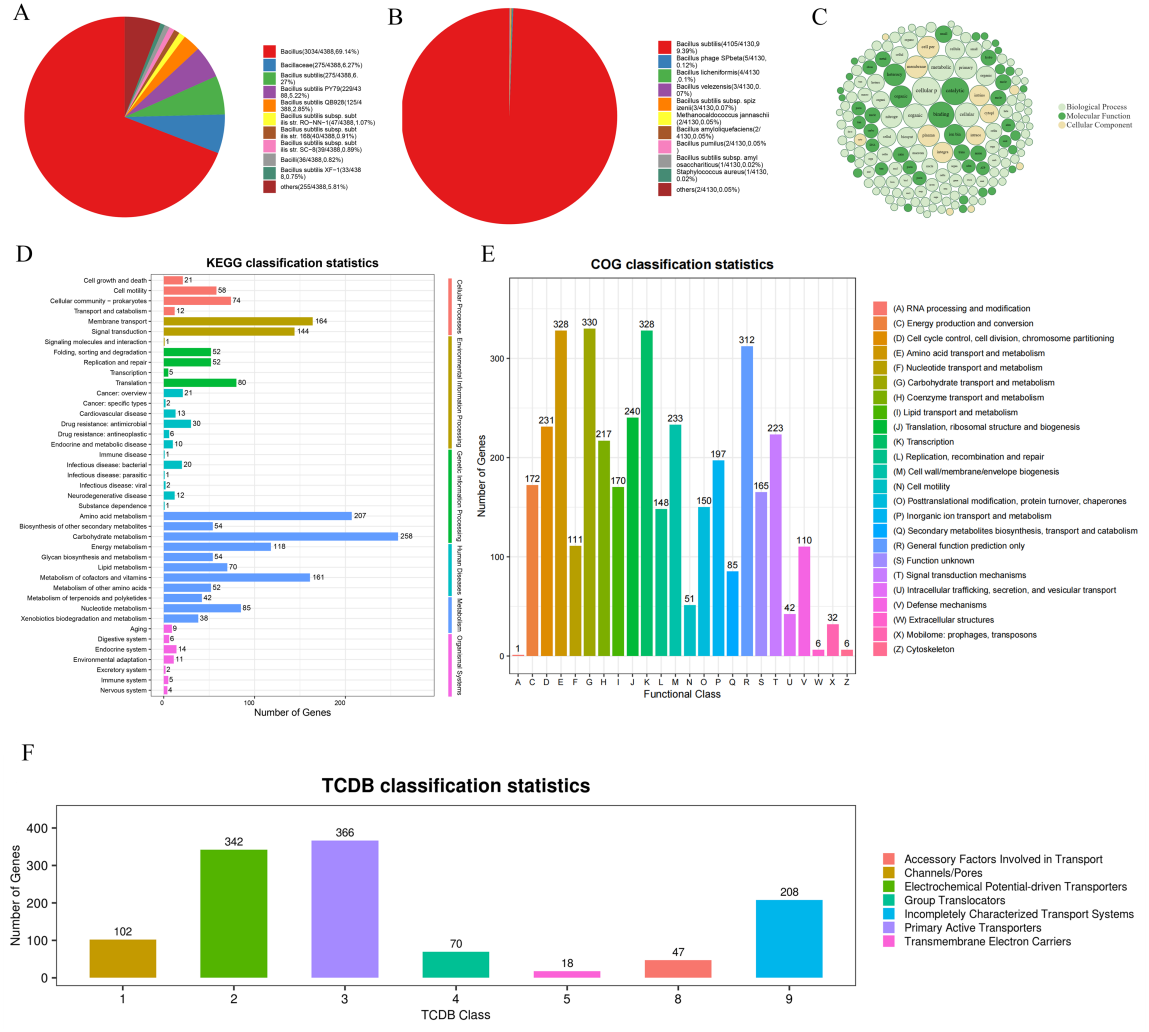
Annotation results of strain JNF2 basic function database. (A) Top10 species distribution map of NR library; (B) SwissProt library Top10 species distribution map; (C) GO annotation results bubble diagram of strain JNF2; (D) The second level of KEGG annotation results of strain JNF2; (E) COG functional classification map of strain JNF2; (F) TCDB classification statistical chart of strain JNF2.

Moreover, the alignment in the NR database show that 69.14% of the sequences are most similar to the sequences of *Bacillus*, 6.27% of the sequences are most similar to the sequences of *Bacillaceae*, 6.27% of the sequences are most similar to the sequences of *B. subtilis*, 5.22% to *B. subtilis* PY79 (Figure 6A). In the SwissProt database, 99.39% of the sequences were most similar to *B. subtilis* (Figure 6B). Based on the above analysis, JNF2 strain was identified as *Bacillus subtilis*.

### 3.6 Genome function annotation and analysis of JNF2 strain

#### 3.6.1 Annotation and analysis of *Bacillus subtilis* JNF2 strain genome basic function database

The NR database alignment results revealed 4,388 predicted coding proteins were predicted in the JNF2 genome (Figure 6A). Comparing with the Swiss-Prot database, a total of 4,130 annotated proteins were identified in the JNF2 genome (Figure 6B).

Analysis of the GO database indicated 3,435 annotated genes in the JNF2 genome with a focus on biological processes such as cellular processes, metabolic processes, biological regulation, localization, and regulation of biological processes. The developmental process is annotated to 1,918, 1,542, 378, 373, 342 and 334, respectively. Notably, 4,336 genes were annotated to molecular functions, including activities in catalytic (1,793), bindings (11,585), transporter (400), transcription regulator (192), and ATP-dependent activities (162). There were 2,413 coding genes annotated to the cellular component, including cellular anatomical entity (2,184), protein-containing complex (223), and virion component (6) (Figure 6C).

In the JNF2 genome sequence, 2,383 genes were annotated with KEGG functions, representing for 53.87% of the total genome. Notably, metabolism-related genes were the most abundant with subcategories such as genes related to carbohydrate metabolism (258), amino acid metabolism (207), metabolism of cofactors and vitamins (161). Additionally, genes related to energy metabolism (118), Nucleotide metabolism (85), lipid metabolism (70), glycan biosynthesis and metabolism (54), and biosynthesis of other secondary metabolism (54), metabolism of other amino acids (52), metabolism of terpenoids and polyketides (42), and xenobiotics biodegradation and metabolism (38) (Figure 6D).

The COG database alignment results showed annotation of 3,376 genes of JNF2 in the JNF2 genome with categories such as Transcription (328), Amino acid transport and metabolism (328), General function prediction only (312), Translation (240), ribosomal structure and biogenesis (Figure 6E).

#### 3.6.2 Genome-specific functional annotation and analysis of JNF2 strain

This study conducted a comprehensive analysis of genome of *B. subtilis* JNF2 reveal a total of 339 annotated resistance genes, which included both antibiotic synthesis and antibiotic resistance genes (Supplementary Table S5). Additionally, 694 genes were identified in the VFDB database, with the quorum-sensing regulatory gene *luxS* (Supplementary Table S6). Furthermore, the PHI database contained annotations for 1,398 genes, of which 942 were associated with reduced virulence, 449 had no impact on pathogenicity, and 127 were linked to enhanced virulence (high virulence) (Supplementary Table S7).

In the TCDB database, 1,153 genes were annotated in the genome of *B. subtilis* JNF2 with specific number of genes identified for various transport such as cofactors involved in transport, channels/pores, electrochemical potential-driven transporters, group translocations, incompletely characterized transport systems, primary active transporters, and protein transmembrane electron carriers (Figure 6F).

### 3.7 Genome CAZymes analysis of JNF2 strain

**Figure 7:**
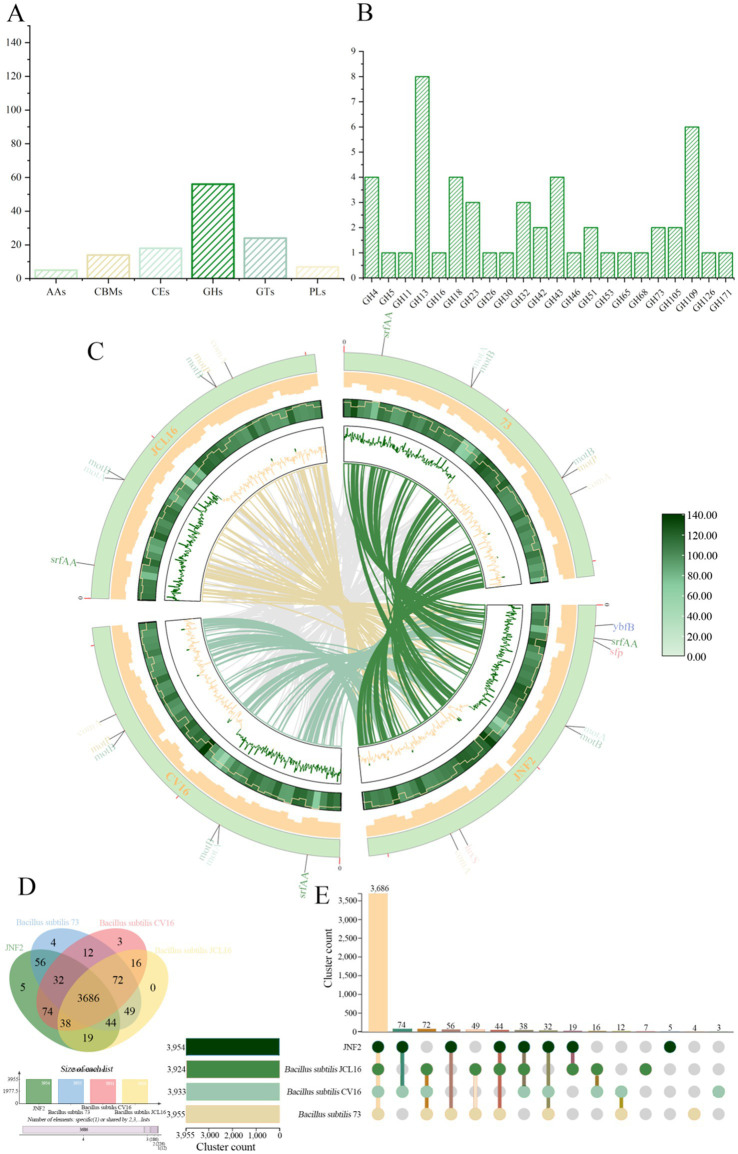
CAZymes analysis of strain JNF2 genome and genome comparison between strain JNF2 and other three strains. (A) Carbohydrate enzyme statistics of *B. subtilis* JNF2 genome; (B) Functional characterization of glycoside hydrolase family of strain JNF2 based on CAZymes; (C) The collinearity analysis circle diagram of JNF2 and the other three strains. The innermost circle was the comparison of multi-strain JNF2 with the other three strains, followed by the n ratio, GC skewness, gene density and GC ratio of the genomes of the four strains; (D) Venn diagram showed the number of unique and common gene clusters between JNF2 and the other three strains; (E) Schematic diagram of homologous gene clusters in different species.

A total of 117 genes were annotated in the JNF2 genome in the CAZymes database. These genes included 56 glycoside hydrolases (GHs), 24 glycosyl transferases (GTs), 18 carbohydrate esterases (CEs), and 14 carbohydrate-binding modules (CBMs). Additionally, seven polysaccharide lyases (PLs) and five auxiliary oxidoreductases (AAs) play a crucial role in facilitating enzyme substrate binding (Figure 7A). Furthermore, seven of them (orf00323, orf01976, orf01979, orf02900, orf03220, orf03327 and orf03724) were classified as both GHs and CBMs simultaneously (Supplementary Table S8). All CAZymes possess an amino acid terminal signal peptide for guiding protein secretion through the cell membrane, indicating their nature as secretory enzymes. The GH family is capable of degrading polysaccharides and disrupting biofilms. Specific antifungal CAZymes within the GH family, such as chitinase (GH18), cellulase (GH5), endoglucanase (GH5, GH51), β-glucanase (GH1), starch debranching enzyme (GH13_28, GH13_29, GH13_31), L-fructosyltransferase (GH32), have the potential in inhibiting plant pathogen growth (Figure 7B). The GH43 family includes enzymes like *α*-L-arabinofuranosidase, β-D-xylosidase, α-L-arabinosidase and β-D-galactosidase that are involved in debranching and degrading hemicellulose and pectin polymers. The GH68 family enzymes act on 2,6-β-D-fructan, 6-β-D-fructosyltransferase, β-fructofuranosidase and inulinase, using sucrose as the preferred donor substrate to produce long levan-type fructan (catalyzed by levansucrases) or inulin-type fructan (catalyzed by inulosucrases) and fructose oligosaccharides (FOS). Additionally, in the absence of sucrose or high fructan/sucrose ratios, some GH68 enzymes can also use fructan as a donor substrate. GH3 enzyme is mainly comprises of stereochemically retained β-glucosidase, with a subfamily of β-N-acetylglucosaminidase that is widely distributed in bacteria, fungi and plants. It has a series of functions such as cellulose biomass degradation, plant and bacterial cell wall remodeling, energy metabolism and pathogen defense. The distribution of CAZymes in the genome of strain JNF2 showed that it had the ability to resist bacteria and fungi.

### 3.8 The secondary metabolic potential of JNF2 strain

Fourteen gene clusters related to the biosynthesis of secondary metabolites were predicted in the whole genome of *B. subtilis* JNF2. These clusters include 4 encoding non-ribosomal peptide synthase (NRPS), 2 encoding terpenes, 2 encoding sactipeptide, 1 encoding RRE-containing, 1 encoding trans-acyl transferase polyketide synthase (transAT-PKS), 1 encoding ranthipeptide, 2 encoding class III polyketides (T3PKS), 1 encoding β-lactone, 1 encoding PKS-like, and 1 encoding glycocin. Additionally, there is 1 cluster encoding NRP-metallophore, 1 encoding CDPS, 1 encoding epipeptide, and 1 that is similar to the bacilysin biosynthetic gene cluster. Among the 4 gene clusters encoding NRPS, 3 had 100% similarity with known synthetic gene clusters of bacillaene, fengycin and bacillibactin (Table 3). Several of these gene clusters have demonstrated antibacterial activity. Furthermore, most of the gene clusters responsible for secondary metabolites in the JNF2 strain are also present in the genomes of the three other strains (Supplementary Table S9).

**Table 3:** Prediction of secondary metabolites in the genome of *B. subtilis* JNF2.

Clusters	Types	Start	End	Most similar known clusters	Similarity
Clusters 1	Sactipeptide, ranthipeptide	203,540	226,493	Sporulation killing factor	100%
Clusters 2	NRPS	356,567	421,958	Surfactin	82%
Clusters 3	Terpene	1,149,542	1,170,345		
Clusters 4	transAT-PKS, PKS-like, T3PKS, NRPS	1,763,368	1,878,126	Bacillaene	100%
Clusters 5	NRPS, betalactone	1,939,803	2,017,562	Fengycin	100%
Clusters 6	Terpene	2,091,774	2,113,672		
Clusters 7	Glycocin	2,259,127	2,279,279	Sublancin 168	100%
Clusters 8	T3PKS	2,296,562	2,337,659	1-carbapen-2-em-3-carboxylic acid	16%
Clusters 9	NRP-metallophore, NRPS	3,260,120	3,311,897	Bacillibactin	100%
Clusters 10	CDPS	3,593,422	3,614,168	Pulcherriminic acid	100%
Clusters 11	Sactipeptide	3,825,686	3,847,297	Subtilosin A	100%
Clusters 12	Other	3,850,296	3,891,714	Bacilysin	100%
Clusters 13	RRE-containing	4,087,776	4,108,045		
Clusters 14	Epipeptide	4,115,368	4,137,066	Thailanstain A	10%

### 3.9 Genome comparison

The collinearity between the genome of *Bacillus subtilis* JNF2 and the other three *Bacillus subtilis* strains was analyzed by the whole genome sequence in the NCBI sequence library. Mauve software was used to identify conserved genomic regions and rearrangement regions in multiple genomes. The experimental results showed that there was a genetic inversion between JNF2 strain and *Bacillus subtilis* 73, *Bacillus subtilis* CV16 and *Bacillus subtilis* JCL16, and gene mutation may occur (Figure 7C).

In this study, JNF2 and three other *Bacillus subtilis* strains (*Bacillus subtilis* 73, *Bacillus subtilis* CV16, *Bacillus subtilis* JCL16) were compared for pan-genomic analysis. The number of homologous genes of JNF2, 73, CV16 and JCL16 was 3,954, 3,955, 3,933 and 3,924, respectively. The number of single-copy genes was 438,160, 182 and 43, respectively (Table 4). The number of homologous genes shared by the four strains was 3,686 and there were 5 specific gene clusters of JNF2. There are 3 specific gene clusters of CV16. There are 4 specific gene clusters of 73. JCL16 has no specific gene cluster (Figures 7D,E).

**Table 4:** The number of proteins, homologous genes, and single copy genes of *B. subtilis* JNF2 and the other three strains of *B. subtilis.*

Species	Protein	Homologous gene	Single-copy gene
JNF2	4,424	3,954	438
*Bacillus_subtilis_*CV16	4,144	3,933	182
*Bacillus_subtilis_*73	4,135	3,955	160
*Bacillus_subtilis_*JCL16	3,984	3,924	43

## 4 Discussion

Cucumber diseases can escalate in severity due to continuous planting and the accumulation of pathogens and pests (Chen et al., 2003). *Fusarium* wilt in cucumbers may result in yield reductions ranging from 15 to 25%, with severe cases leading to losses of up to 50% or even complete loss of yield (Chung et al., 2008). In recent years, plant growth-promoting rhizospheric bacteria (PGPR), particularly *Bacillus* species, have gained recognition for their ability to enhance plant growth. *Bacillus subtilis* not only produces endophytic spores but also synthesizes various antibiotics and indoleacetic acid (Xia et al., 2007). Research by Faltin et al. found that plant hormones produced by *Bacillus subtilis* significantly promoted lettuce leave growth using plate micropore method (Faltin et al., 2004). The cell wall of fungi is complex, consisting mainly of components include cellulose, chitin, glucan and protein (Shen et al., 2017). Biocontrol experiments of cucumber plants in this study revealed that strain JNF2 produced indole-3-acetic acid (IAA) and siderophore, along with β-1,3-glucanase, protease and amylase activities. IAA, a crucial plant hormone, regulates growth rates and promotes rooting. Iron, essentially for plant growth and development, plays a significant role in plant metabolism and biochemical reactions. Therefore, JNF2 not only disrupts the cell wall of pathogenic fungi through hydrolases but also stimulates plant growth by producing IAA and siderophores, shedding light on its antibacterial and growth-promoting mechanisms. Pot experiments showed that cucumber seedlings inoculated with both *FOC* and JNF2 strains outperformed those inoculated with only *FOC*, exceeding the growth-promoting effects of the chemical fungicide Hymexazol. Thus, strain JNF2 shows promise as a biocontrol agent.

Under an electron microscope, the mycelium of the pathogen exhibited abnormal shrinkage following antagonism, potentially due to metabolites produced by strain JNF2 that disrupted mycelial cells, leading to leakage of cellular contents. In conclusion, the JNF2 demonstrates a significant inhibitory effect on *FOC*-FJH36, causing abnormal morphology in the pathogen.

Plant disease resistance involves complex interplay of physiological and biochemical changes, with defense enzymes such as LOX, CAT, PAL, SOD, PPO playing important regulatory roles (Zhou et al., 2020). SOD, for instance, acts as a free radical scavenger in plants, maintaining the balance of reactive oxygen species when plants during stress (Ma and Zhu, 2003). In measuring defense enzyme activity, cucumber seedlings inoculated with strain JNF2 exhibited significantly higher activities of LOX, PAL, CAT, PPO and SOD compared to the control group. Interestingly, while SOD levels were slightly lower in the JNF2-treated seedlings, the activities of other defense enzymes were higher in this group, possibly due to the reduced stress levels in plants inoculated with JNF2. These findings suggest that the inoculation of strain JNF2 can enhance resistance in the plants infected with *FOC*.

The genome sequencing of strain JNF2 showed a composition of 4,215,642 bp in circular chromosomes with GC content of 43.51%. Comparison of 16S rRNA gene sequence and phylogenetic tree analysis indicated a close genetic relationship with *Bacillus subtilis*. Furthermore, ANI analysis showed that the ANI values between strain JNF2 and *B. subtilis* 73, *B. subtilis* JCL16 and *B. subtilis* CV16 were all above 98%, suggesting that JNF2 belonged to *Bacillus subtilis*. Using Mauve software, the collinearity analysis of the four strains showed that high homology between the strain JNF2 and CV16 and 73, with a possible horizontal gene transfer with JCL16. Notably, strain JNF2 encoded by a great number of proteins and gene clusters compared to that of the other three strains, indicating potential functional specificity, and a distinct biocontrol mechanism within the *Bacillus subtilis* strains. However, the study did not investigate whether the specific gene cluster of strain JNF2 could produce related compounds, and highlighting the need for further experimental research.

Biofilm refers to a microbial community that attaches to the surface of the object with microorganisms living in the extracellular polymers they produce (O’Toole et al., 2000). There are also abundant microbial communities around the nutrients in the rhizosphere. Studies have shown the importance of rhizosphere microbial biofilms in the preventing and treating soil-borne diseases. For instance, *Bacillus subtilis* 6,051 can form a stable and extensive biofilm after colonization in the roots of *Arabidopsis thaliana*, protecting them from the invasion of *Syringa pseudomonads* (Bais et al., 2004). Various environmental factors influence the colonization of biocontrol bacteria in plant roots, which is strictly regulated by the bacteria system (Miller and Bassler, 2001). Among them, the quorum sensing system is a mechanism by which bacteria regulate gene expression by detecting the threshold of signal molecules produced by themselves. This system can regulate a variety of physiological activities including biofilms. At present, the QS system with autoinducer 2 (AI-2) as a signal molecule is one of the quorum sensing systems (Rezzonico et al., 2012). The *luxS* gene can generate *LuxS*. *LuxS* catalyzes S-ribose homocysteine and 4,6-hydroxy-2,3-pentanedione (DPD) to produce AI-2 spontaneously (Han and Lu, 2009). Therefore, *luxS* is an important gene in the quorum sensing system. In the genome structure annotation of the strain JNF2 in this study, the known genes related to biofilm formation like *luxS*, *motA* are identified. Overall, strain JNF2 has the potential to colonize cucumber plant roots to form biofilms to resist the invasion of *FOC*-FJH36.

The analysis of the JNF2 strain genome through CAZymes reveals that 117 genes are annotated in the CAZymes database, with 56 of them being glycoside hydrolases (GHs). Research indicates that the GH family contains antifungal components like chitinase and cellulase. This conclusion further elaborates that the JNF2 strain has the potential to inhibit the growth of pathogens.

Furthermore, glycocins, which are glycosamino acid-containing bacteriocins containing glycocin F from *Lactobacillus plantarum* has been showed to inhibit bacteria like *Streptococcus pyogenes* and *Listeria monocytogenes* (Teng and Zhong, 2022). This study also reveals that *B. subtilis* JNF2 harbors a synthetic gene cluster for glycocin, which is absent in other strains like *B. subtilis* 73, *B. subtilis* JCL16, and *B. subtilis* CV16. Therefore, these differences in the synthetic gene clusters between *B. subtilis* JNF2 and the other three strains may impact their ability to combat plant diseases.

Additionally, secondary metabolism analysis of *B. subtilis* JNF2 indicates the presence of NRPS, transAT-PKS and other synthetic gene clusters. The most widely studied active substance of *B. subtilis* is lipopeptide antibiotics synthesized through the NRPS pathway. Arima et al. first discovered the antibacterial lipopeptide surfactin in *B. subtilis* (Arima et al., 1968). At present, more lipopeptides synthesized by NRPS pathway have been found in *B. subtilis*, such as fengycin family (Brakhage and Schroeckh, 2011), NRPS iron carrier (bacillibactin) with significant antibacterial effects (Challis and Ravel, 2000). Ongena et al. proved that surfactin and fengycin in *B. subtilis* can induce defensive responses in soybean through the salicylic acid (SA) pathway (Ongena et al., 2002). Moreover, PKS pathway in *B. subtilis*, is responsible for synthesizing a range of polyketides including bacillaene, difficidin, and macrolactin antibiotics (Hamdache et al., 2011). Among them, bacillaene is a polyene antibiotic, which is synthesized by tran-AT PKS in type I PKS (Moldenhauer et al., 2007). This substance can antagonize bacteria and fungi by inhibiting the synthesis of related proteins (Um et al., 2013). Therefore, *B. subtilis* JNF2 can not only produce corresponding antibiotics to antagonize pathogens, but also trigger disease resistance by secreting related substances.

Through genome-wide analysis of *B. subtilis* JNF2, it was discovered that the strain harbored numerous genes associated with siderophore biosynthesis, including *feuA, feuB* and *feuC*, thereby confirming its ability to produce siderophore and promote plant growth. Secondary metabolism analysis revealed the presence of surfactin, fengycin and other synthetic gene clusters in *B. subtilis* JNF2. Previous studies by Lopez et al. demonstrated that surfactin in *B. subtilis* model strain 3,610 functions as a quorum sensing signal molecule inducing biofilm formation through interaction with the membrane protein kinase KinC (Lopez et al., 2009). This dual role of surfactin as an antibacterial lipopeptide surfactant and promotor of biofilm synthesis effectively prevents pathogen infections.

**Figure 8:**
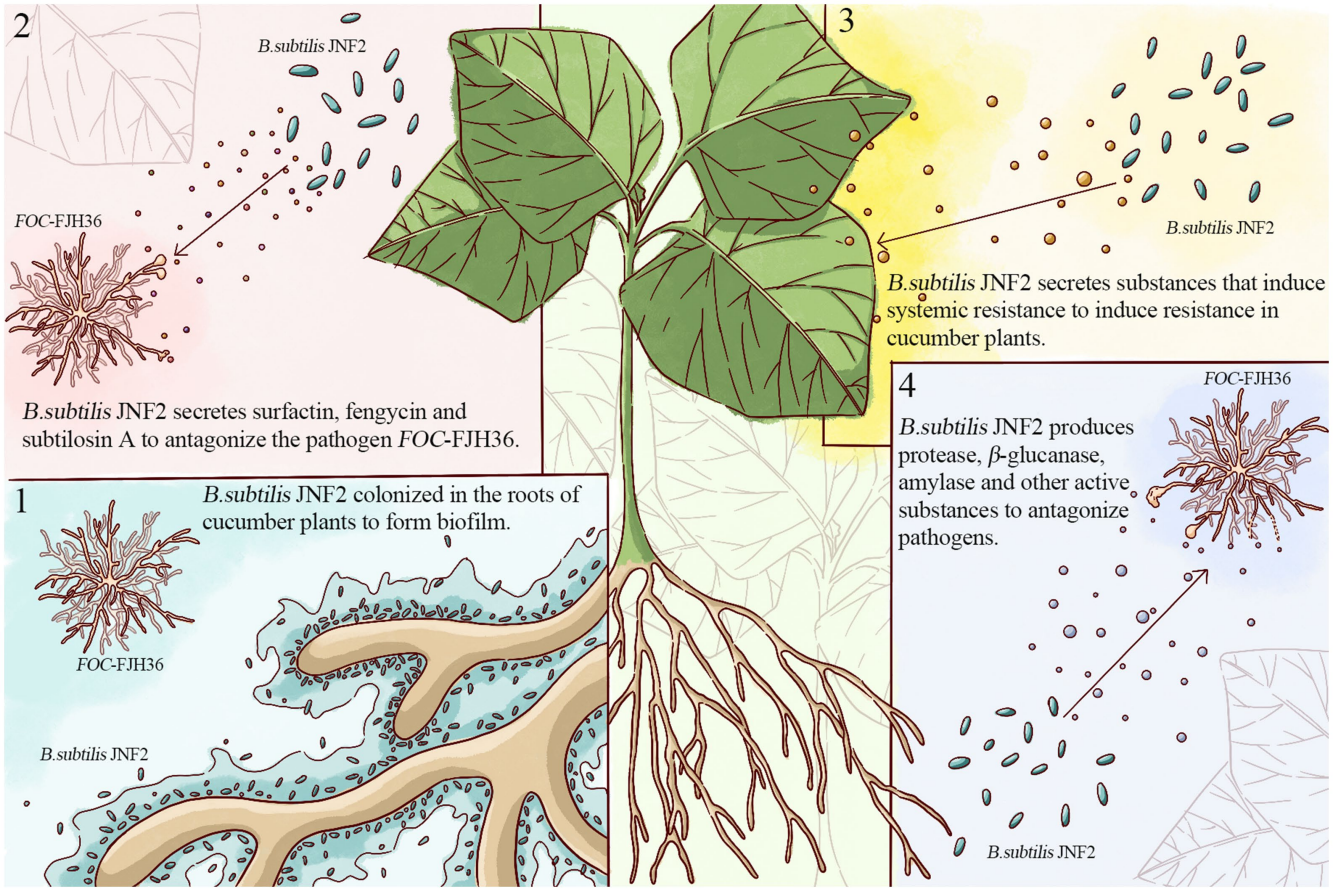
Prediction of four biocontrol mechanisms of *Bacillus subtilis* JNF2.

In conclusion, this study identified JNF2 strain as *Bacillus subtilis* and elucidated its biocontrol mechanism (Figure 8), involving in root colonization to form biofilms, secretion of antibiotics against the pathogen *FOC*-FJH36, induction of systemic disease resistance in cucumber plants, and production of proteases and active substances to support JNF2 proliferation and plant colonization. Thus, the control effect of cucumber *Fusarium* wilt is achieved through nutrition competition and niche competition. While *B. subtilis* JNF2 demonstrated antagonistic effects against pathogenic bacteria and reduced mycelium growth, further experiments were needed to further explore the mechanism of abnormal state of mycelium by transmission scanning electron microscopy. In addition, it can be inferred from the experimental results that there may be some kind of communication signal between *B. subtilis* JNF2 and the host, which promotes the bacteria to form a biofilm in the roots of cucumber plants. Therefore, the signal molecules of the signal pathway between *B. subtilis* JNF2 and host plants and the molecular mechanism of *B. subtilis* recognition can be studied in the future to understand the related mechanism of *B. subtilis* biocontrol diseases.

## Data Availability

The datasets presented in this study can be found in online repositories. The names of the repository/repositories and accession number(s) can be found in the article/Supplementary material.
